# Evidence supporting the choice of a new cardiovascular risk equation for Australia

**DOI:** 10.5694/mja2.52052

**Published:** 2023-07-26

**Authors:** Sinan Brown, Emily Banks, Mark Woodward, Natalie Raffoul, Garry Jennings, Ellie Paige

**Affiliations:** ^1^ National Centre for Epidemiology and Population Health Australian National University Canberra ACT; ^2^ The George Institute for Global Health University of New South Wales Sydney NSW; ^3^ The George Institute for Global Health Imperial College London London United Kingdom; ^4^ National Heart Foundation of Australia Sydney NSW; ^5^ University of New South Wales Sydney NSW; ^6^ QIMR Berghofer Medical Research Institute Brisbane QLD

**Keywords:** Absolute risk, Guidelines as topic

## Abstract

- This article reviews the risk equations recommended for use in international cardiovascular disease (CVD) primary prevention guidelines and assesses their suitability for use in Australia against a set of a priori defined selection criteria.
- The review and assessment were commissioned by the National Heart Foundation of Australia on behalf of the Australian Chronic Disease Prevention Alliance to inform recommendations on CVD risk estimation as part of the 2023 update of the Australian CVD risk assessment and management guidelines.
- Selected international risk equations were assessed against eight selection criteria: development using contemporary data; inclusion of established cardiovascular risk factors; inclusion of ethnicity and deprivation measures; prediction of a broad selection of fatal and non‐fatal CVD outcomes; population representativeness; model performance; external validation in an Australian dataset; and the ability to be recalibrated or modified.
- Of the ten risk prediction equations reviewed, the New Zealand PREDICT equation met seven of the eight selection criteria, and met additional usability criteria aimed at assessing the ability to apply the risk equation in practice in Australia.

Cardiovascular disease (CVD) is the leading cause of death globally, responsible for an estimated 17.9 million deaths in 2019,^1^ and accounting for 25% of all deaths in Australia in 2021.^2^ CVD is highly preventable through health risk behaviour modification and pharmacotherapy. Assessment and treatment of cardiovascular risk using validated risk equations is considered international best practice and is the cornerstone of primary CVD prevention.

In the 2012 version of the Australian guidelines on CVD risk assessment and management,^3^ the Framingham risk equation^4^ was recommended as part of an overall algorithm that included a first stage assessment of an individual's medical history.^3^ The United States Framingham equation was the first and is still the best known equation for assessing absolute CVD risk.^5^ Although pioneering at the time, its development in a predominantly white US cohort recruited from 1948 limits its applicability in contemporary Australia. On average, it overestimates risk for the general Australian population^6^ and underestimates risk for Aboriginal and Torres Strait Islander peoples in both remote and urban settings.^7^, ^8^

Many countries, including New Zealand, the United Kingdom, the US and several European countries have developed country‐ or region‐specific risk equations based on local data and tailored to local CVD event rates. Australia currently lacks the large scale contemporary linked datasets with population‐based data on CVD risk factors and event rates to generate an Australian‐specific CVD risk equation. An alternative is to use an existing risk equation and recalibrate it to align with the CVD event rates observed in Australia.

There is little published guidance on how to choose an existing CVD risk equation for a country. In 2020, the World Health Organization (WHO) published a technical package (HEARTS) for risk‐based CVD management in primary health care, which included a process for selecting a risk equation.^9^ However, this primarily focused on the use of either population‐specific risk equations or the WHO global CVD risk charts and does not consider the use of other existing risk equations.

The Australian CVD risk assessment and management guideline, including the risk equation, was updated in 2023 (https://www.cvdcheck.org.au). This article outlines the process and evidence informing the decision on the CVD risk equation recommended for Australia in the 2023 guideline update.

## Methods

We used a systematic approach to evaluate the appropriateness of existing international CVD risk equations for use in primary care in Australia. First, selection criteria were developed to guide the choice of the most appropriate risk equation. Second, we undertook a review of existing international CVD risk equations, focusing on risk equations currently recommended in major international guidelines. Third, the existing international risk equations identified through the review were assessed against each selection criterion. Evidence from this review and comparison to selection criteria were presented to the Guideline Expert Steering Group to inform decisions on the risk equation recommended for Australia in the updated CVD risk assessment and management guidelines.

A set of a priori selection criteria were defined at a National Stakeholder Roundtable on CVD risk, hosted by the National Heart Foundation of Australia and the Australian National University. The Roundtable was held on 19 November 2019 and included 24 attendees from 13 organisations: the National Heart Foundation of Australia, the Australian National University, Monash University, the University of Sydney, the University of Queensland, Northern Hearts WA, Sunshine Coast University, Bond University, the University of Tasmania, The George Institute for Global Health, the University of Adelaide, the Commonwealth Scientific and Industrial Research Organisation, and the University of Auckland. The Roundtable agreed on the following criteria for the selection of a new CVD risk equation for Australia:

- use of contemporary data — because the prevalence of risk factors and the relationship of predictors to CVD outcomes can change over time, risk equations based on more contemporary datasets are likely to be better at predicting risk for that population;
- inclusion of established CVD risk factors — age, sex, cholesterol, blood pressure, diabetes and smoking;
- consideration of measures of ethnicity and social deprivation (to improve health equity);
- inclusion of a broad range of CVD events and deaths as outcomes, such as myocardial infarction, stroke and coronary heart disease;
- population representativeness, either of the general population or the primary care population;
- excellent model performance including discrimination (C‐statistic > 0.7) and calibration in the population in which the equation was tested;
- external validation in an Australian population; and
- ability to be recalibrated and modified for a different population.

A targeted narrative review of major international CVD risk management guidelines and the risk equations recommended by the Roundtable was conducted in February 2020, supplemented by a previous literature review on CVD risk equations **(**Buttery AK, Matthews S, Walton N. Overview of international risk prediction models used for the primary prevention of cardiovascular events [unpublished article]. National Heart Foundation of Australia, 2021). We reviewed the most recent CVD risk assessment guidelines from Australia, NZ, Canada, US, UK, Scotland, Europe, Norway, Japan, and the WHO. For each guideline, information was extracted on the eligible/target population, clinically determined high risk criteria, recommended risk equation, risk categories and time frame, the pharmacotherapy risk treatment threshold, and the frequency of risk assessment.

For each unique CVD risk equation recommended in one or more of the selected guidelines, we extracted detailed information on: development dataset and population; sample size; CVD outcome definitions and time frames of risk estimation; age range of participants in the development dataset; variables included in the equation; details on model performance (discrimination) in the development dataset and in Australian validation datasets (where applicable); population representativeness; and a list of countries where the equation has been validated. Only risk equations which estimated CVD risk over a given time period (ie, not lifetime risk) were assessed.

Each of the risk equations recommended in the guidelines was then assessed against the selection criteria, noting whether each criterion had been met. For CVD risk equations that met most of the selection criteria, practical implications of applying the risk equation in Australia were considered, guided by two of the usability considerations outlined in the WHO HEARTS technical package,^9^ specifically whether: (i) the format (online risk calculator, charts) available for the risk equation was appropriate for the target population; and (ii) the risk factors could be feasibly measured in the target population.

## Review of existing international CVD guidelines and risk equations

Eleven international guidelines on CVD risk management were identified, five of which were from Europe (Box 1). There was wide variation in which underlying CVD risk equations were recommended, with a total of 12 unique CVD risk equations recommended for use, 10 of which estimated risk over a specific time period (Box 2). All but two estimated risk over a 10‐year period, most were derived from general or cohort population samples, and all but one had been validated in one or more external datasets (Box 3). Although the QRISK2 risk equation is recommended for use in UK guidelines, we also assessed the QRISK3 risk equation which supersedes QRISK2 and is currently available for use in primary care practices in the UK.

Box 1 **Summary of main international guidelines for the prevention of cardiovascular disease**

**Table:** 

Guideline name	Country/region	Published/updated	Developed by	Comments/developments
Guidelines for the management of absolute cardiovascular disease risk^3^, ^10^	Australia	2012	National Vascular Disease Prevention Alliance (NVDPA)	Updates previous NVDPA guidance (2009)
2016 Canadian Cardiovascular Society guidelines for the management of dyslipidemia for the prevention of cardiovascular disease in the adult^11^, ^12^, ^13^	Canada	2016	Canadian Cardiovascular Society (CCS)	Updates previous CCS guidance (2013) New guidelines published (2021)
Cardiovascular disease risk assessment and management for primary care^14^, ^15^	New Zealand	2018	New Zealand Ministry of Health	Updates 2012 Primary Care Handbook
Cardiovascular disease: risk assessment and reduction, including lipid modification^16^, ^17^, ^18^	England and Wales	Published 2014; updated 2016	National Institute for Health and Care Excellence (NICE)	Previous guidelines published 2008
Joint British Societies’ consensus recommendations for the prevention of cardiovascular disease (JBS3)^19^, ^20^	United Kingdom	2014	British Cardiovascular Society (BCS)	Updates JBS2 (2005)
Risk estimation and the prevention of cardiovascular disease^21^	Scotland	2017	Scottish Intercollegiate Guidelines Network (SIGN)	Updates previous SIGN guidance (2007)
2016 European guidelines on cardiovascular disease prevention in clinical practice^22^, ^23^, ^24^	Europe	2016	The Sixth Joint Task Force of the European Society of Cardiology and Other Societies on Cardiovascular Disease Prevention in Clinical Practice	Updates previous European guidelines (2012) New guidelines published (2021)
New guidelines for the prevention of cardiovascular disease^25^	Norway	2017	Norwegian Directorate of Health	Updates previous national guidelines (2009)
2019 ACC/AHA guideline on the primary prevention of cardiovascular disease^26^, ^27^	United States	2019	American College of Cardiology/American Heart Association (ACC/AHA) Task Force on Clinical Practice Guidelines	Updates previous ACC/AHA guidelines (2013)
Japan Atherosclerosis Society (JAS) guidelines for prevention of atherosclerotic cardiovascular diseases 2017^28^	Japan	2018	Japan Atherosclerosis Society (JAS)	Guidelines published every five years
Prevention of cardiovascular disease: guidelines for assessment and management of cardiovascular risk^29^, ^30^	Global	2007	World Health Organization (WHO)	Guidelines developed on basis of total risk approach, elaborated in World Health Report (2002)

Box 2 **Overview of risk assessment algorithms recommended in guidelines**

**Table:** 

Country/region	Eligible population	Clinically determined high risk categories	Risk equation name	Risk categories and time frame	Pharmacotherapy risk treatment threshold	Risk assessment frequency
Australia^3^, ^4^	Adults ≥ 45 years (≥ 35 years for Aboriginal and Torres Strait Islander peoples) without previous history of CVD	DM and age > 60 years, DM with microalbuminuria, moderate/severe CKD, familial hypercholesterolaemia, high cholesterol, high BP, Aboriginal and Torres Strait Islander peoples > 74 years	Framingham–Anderson (1991)	5‐year risk: high (> 15%); moderate (10–15%); low (< 10%)	High risk Not routinely recommended for low/moderate risk, unless BP, family history or sub‐population criteria met	Timing of absolute risk review varies depending on risk level (every 2 years for low risk, 6–12 months for moderate risk, and “according to clinical context” for high risk)
Canada^12^, ^31^, ^32^	Adults 40–75 years	Clinical atherosclerosis, abdominal aortic aneurysm, DM, CKD, hypercholesterolaemia (including genetic dyslipidaemias)	Cardiovascular Life Expectancy Model (CLEM) or Framingham–D'Agostino (2008)	Using Framingham–D'Agostino (2008), 10‐year risk: high (≥ 20%); intermediate (10–19%); low (< 10%)	High risk Consider in low/intermediate risk if additional criteria met	Every 5 years
New Zealand^14^, ^33^	Adults 30–74 years without pre‐existing CVD; begin in men ≥ 45 years, women ≥ 55 years; for Māori, Pacific and South‐Asian peoples, begin in men ≥ 30 years, women ≥ 40 years	Prior CVD event, CHF, DM with overt nephropathy or other renal disease, CKD, familial hypercholesterolaemia	NZ Primary Prevention Equations/PREDICT	5‐year risk: > 15%; 5–15%; < 5%	Strongly recommended if > 15% risk Considered (benefits and harms discussed) if 5–15% risk	Timing of repeat assessment varies depending on risk level (every 10 years for < 3% risk and annually for > 15% risk)
England and Wales^16^, ^17^, ^34^, ^35^	Adults ≤ 84 years without pre‐existing CVD, type 1 DM, familial hypercholesterolaemia or other inherited disorders of lipid metabolism	eGFR < 60 mL/min/1.73 m^2^	QRISK2; QRISK3 derived and validated (2017), yet to be incorporated into guidelines	10‐year risk	Atorvastatin offered to those with a 10% or greater risk	Adults > 40 years should have their absolute risk estimate reviewed on an ongoing basis
United Kingdom^19^, ^36^	All adults except those with existing CVD or other high risk conditions (as listed)	Established CVD, DM and age > 40 years, CKD, familial hypercholesterolaemia	QRISK Lifetime	Lifetime risk	Threshold for statin treatment informed by NICE guidelines (2014): 10% risk	
Scotland^21^, ^37^	All adults ≥ 40 years, adults at any age with first‐degree relative with premature atherosclerotic CVD or familial dyslipidaemia	Established CVD, DM and age > 40 years, DM and age < 40 years with presence of other risk factors (eg, target organ damage), CKD, familial hypercholesterolaemia, micro‐ or macroalbuminuria	ASSIGN	High (≥ 20% over 10 years); must also be asymptomatic and without established CVD (or other conditions which confer automatically high cardiovascular risk)	High risk adults without established CVD should be offered atorvastatin	At least once every five years if > 40 years and without history of CVD, familial hypercholesterolaemia, CKD or DM and not being treated to reduce BP or lipids
Europe^23^, ^38^	Adults > 40 years at increased CV risk (due to family history, familial hyperlipidaemia or other major risk factors) and without established CVD	Documented CVD (clinical or unequivocal on imaging), DM, moderate/severe CKD, markedly elevated single risk factors (eg, cholesterol or BP)	Systematic Coronary Risk Estimation (SCORE)	10‐year risk: very high (≥ 10%); high (≥ 5% to <10%); moderate (≥1% to < 5%); low (< 1%)	High risk adults are candidates	Repeat assessment every 5 years and more often for adults approaching treatment thresholds
Norway^25^, ^39^	Adults 40–79 years Note: age range not mentioned in guidelines but equation developed using data for people aged 40–79 years	Individual factors/criteria can trigger pharmacotherapy recommendation independent of calculated risk: LDL‐C, TC, diastolic BP, systolic BP, hypertension‐induced end‐organ damage, type 1 or 2 DM and age > 40 years	NORRISK 2	10‐year risk: 45–54 years: high (≥ 5.0%); intermediate (4.0–4.9%); low (< 4.0%) 55‐64 years: high (≥ 10.0%); intermediate (8.0–9.9%); low (< 8.0%) 65–74 years: high (≥ 15.0%); intermediate (12.0–14.9%); low (< 12.0%)	Age‐specific intervention thresholds: 45–54 years, ≥ 5%; 55–64 years, ≥ 10%; 65–74 years, ≥ 15%	
United States^26^, ^27^, ^40^	Adults 40–75 years without DM	Familial hypercholesterolaemia	Pooled Cohort Equations	10‐year risk: high (≥ 20%); intermediate (≥ 7.5% to < 20%); borderline (5% to < 7.5%); low (< 5%)	High risk: initiate Intermediate risk: initiate if risk‐enhancing factors are present, eg, family history, CKD, ethnicity	
Japan^28^, ^41^	Adults 35–74 years without history of CHD	Peripheral artery disease, non‐cardiogenic cerebral infarction, DM (excluding impaired glucose tolerance), CKD	Suita score	10‐year risk: high (≥ 9%); moderate (2% to < 9%); low (< 2%)	Pharmacotherapy considered for all risk categories (including low risk) if 3–6 months of behaviour modification ineffective	
Global^29^, ^30^, ^42^	Adults without CHD, stroke or other atherosclerotic disease	Established CVD, DM with overt nephropathy or other significant renal disease, high cholesterol, high BP, renal failure/impairment	WHO/ISH risk charts (2007) Updated charts derived and validated (2019), yet to be incorporated into guidelines	10‐year risk: very high (> 30%); high (20–30%); moderate (10–20%); low (< 10%)*	Statins: very high risk; high risk if dietary changes inadequate/high serum cholesterol Antihypertensive/antiplatelet: very high risk; consider in high risk if behavioural strategies inadequate	

BP = blood pressure; CHD = coronary heart disease; CHF = congestive heart failure; CKD = chronic kidney disease; CVD = cardiovascular disease; DM = diabetes mellitus; eGFR = estimated glomerular filtration rate; ISH = International Society of Hypertension; LDL‐C = low‐density lipoprotein cholesterol; NICE = National Institute for Health and Care Excellence; TC = total cholesterol; WHO = World Health Organization.

* Based on previous 2002 WHO risk charts;^30^ the latest 2019 WHO global risk charts^42^ do not specify risk thresholds.

Box 3 Overview of methods used in the main cardiovascular disease (CVD) risk equations for primary care*

**Table:** 

	Framingham–Anderson (1991)	Framingham–D'Agostino (2008)	PREDICT	QRISK2/QRISK3	ASSIGN	SCORE	NORRISK 2	Pooled Cohort Equations	Suita score	WHO risk charts (2019)
Development datasets	Framingham Heart Study and Framingham Offspring Study	Framingham Heart Study and Framingham Offspring Study	PREDICT cohort study	QResearch database; QRISK2, version 19; QRISK3, version 41	Scottish Heart Health Extended Cohort (SHHEC)	12 European cohort studies from 11 countries	Cohort of Norway (CONOR) study; Cardiovascular Disease in Norway (CVDNOR) project	Atherosclerosis Risk in Communities; Coronary Artery Risk Development in Young Adults; Cardiovascular Health Study; Framingham Heart Study; Framingham Offspring Study	Suita cohort study	Emerging Risk Factors Collaboration (ERFC)
Population	General population in Framingham, US; baselines: 1968–1975	General population in Framingham, US; baselines: 1968–1971; 1971–1975; 1984–1987	NZ primary care patients with PREDICT data; assessment: 2002–2015	Routinely collected general practice data in England (and Wales for QRISK2) QRISK2: 531 practices, 1993‐2008 QRISK3: 1309 practices, 1998–2015	Random‐sample population surveys across Scotland (1984–1987) and North Glasgow (1989, 1992, 1995)	Combination of random sample of general population, occupational and male‐only cohorts; baselines: 1967–1991	CONOR (various regional health surveys): 1994–2003 CVDNOR (database of CVD discharge diagnoses, CHD/stroke mortality): 1994–2009	Several community‐based prospective cohort studies (US); baselines: 1968–1993	Random sample of residents in Suita, Japan; baselines: 1989–1994	85 prospective cohorts (89% individuals from Europe and North America); baselines: 1960–2013
Sample size (total, including any derivation and internal validation samples)	5573 (2590 men, 2983 women)	8491 (3969 men, 4522 women)	401 752 (226 053 men, 175 699 women)	QRISK2: 2 285 815 (1 136 761 men, 1 149 054 women) QRISK3: 10 561 101 (5 180 688 men, 5 380 413 women)	13 297 (6540 men, 6757 women)	205 178 (117 098 men, 88 080 women)	66 712 (31 445 men, 35 267 women)	24 626 (10 745 men, 13 881 women)	5521 (2796 men, 2725 women)	376 177 (202 962 men, 173 215 women)
Outcome definition	4‐ to 12‐year risk of CVD (including CHF, PVD), CVD death, CHD (including angina, coronary insufficiency), CHD death, MI, stroke (including transient ischaemia) Note: in Australia, 5‐year risk is estimated	10‐year risk of CVD event; CHD (angina, coronary insufficiency, coronary death, MI), stroke (haemorrhagic, ischaemic, TIA), heart failure, PAD	5‐year risk of hospitalisation or death from CHD (including unstable angina), stroke (haemorrhagic or ischaemic), TIA, CHF, PVD	10‐year risk of CHD (angina, MI), ischaemic stroke or TIA	10‐year risk of CVD death or hospital discharge diagnosis for CHD, cerebrovascular disease or coronary artery interventions (CABG, PTCA)	10‐year risk of fatal atherosclerotic event, CHD (angina, MI), stroke (haemorrhagic, ischaemic, TIA), heart failure, PAD, PVD	10‐year risk of CHD death, MI, fatal or non‐fatal acute stroke	10‐year risk of hard atherosclerotic CVD event (CHD death, non‐fatal MI, fatal or non‐fatal stroke)	10‐year risk of CHD (MI, sudden cardiac death, coronary revascularisation)	10‐year risk of fatal or non‐fatal CVD event, CHD, MI, stroke
Age range (years)	30–74	30–74	30–74	QRISK2: 35–74 QRISK3: 25–84	Original: 30–74 Current: 25–90	40–65	40–79	40–79	Original: 30–79 Current: 35–74	40–80
Variables included in original risk equation	Age, sex, smoking (cigarette smoker or quit within past year), TC, HDL‐C, DBP or SBP, DM (receiving treatment), LVH Note: in Australia, SBP is used in the equation	Traditional model: age, sex, smoking, HDL‐C, TC, SBP, DM, treated hypertension Non‐laboratory model: office‐based equation which substitutes HDL‐C and TC with BMI	Age, sex, ethnicity, family history, deprivation, smoking (never, ex‐, current), TC:HDL‐C, SBP, T1DM or T2DM, AF, medication prescription (BP, lipid, antithrombotic)	QRISK2: age, sex, ethnicity, family history, deprivation, smoking (non‐smoker [including ex‐], current), BMI, TC:HDL‐C, SBP, T2DM, AF, RA, CKD, treated hypertension QRISK3: all of the above, plus: smoking (non, former, light, moderate, heavy), BP variability, T1DM, SLE, severe mental illness, migraine, erectile dysfunction, atypical antipsychotics, corticosteroids	Age, sex, family history, deprivation, smoking (cigarettes per day), HDL‐C, TC, SBP, DM	Age, sex, smoking (current or non‐smoker), TC or TC:HDL‐C, SBP	Age, sex, family history, smoking (daily), HDL‐C, TC, SBP, present use of antihypertensive drugs	Age, sex, ethnicity, smoking (smoker or non‐smoker), HDL‐C, TC, SBP (treated or untreated status), DM	Age, sex, smoking (current or non‐current), HDL‐C, LDL‐C or TC, DBP, SBP, DM, CKD	Laboratory model: age, sex, smoking (current or other), TC, SBP, DM Non‐laboratory model: TC and DM substituted by BMI
Changes to variables in updates to risk equation					New equation includes RA				Family history, impaired glucose tolerance added CKD, DM removed — considered high risk conditions	
Model performance	No model performance statistics reported	Traditional model Women: C‐statistic, 0.79 (0.77–0.81) Men: C‐statistic, 0.76 (0.75–0.78)	Women: C‐statistic, 0.73 (0.72–0.73) Men: C‐statistic, 0.73 (0.72–0.73)	QRISK2: women: AUROC, 0.82 (0.81–0.82); men: AUROC, 0.79 (0.79–0.79) QRISK3: women: C‐statistic, 0.88 (0.88–0.88); men: C‐statistic, 0.86 (0.86–0.86)	Women: AUROC, 0.77 Men: AUROC, 0.73	TC (AUROC) High risk region, 0.81 (0.80–0.82) Low risk region, 0.74 (0.72–0.76) TC:HDL‐C (AUROC) High risk region, 0.80 (0.78–0.82) Low risk region, 0.75 (0.73–0.77)	Women: AUROC, 0.84 (0.83‐0.85) Men: AUROC, 0.79 (0.79‐0.80)	Women: white: C‐statistic, 0.81; African American: C‐statistic, 0.82 Men: white: C‐statistic, 0.75; African American: C‐statistic, 0.71	Performance without CKD variable TC: C‐statistic, 0.83; LDL‐C: C‐statistic, 0.83	Laboratory model Women, C‐index: fatal or non‐fatal MI or CHD death, 0.76 (0.75–0.77); fatal or non‐fatal stroke, 0.74 (0.74–0.75) Men, C‐index: fatal or non‐fatal MI or CHD death, 0.70 (0.68–0.69); fatal or non‐fatal stroke, 0.73 (0.72–0.74)
External validation (cohort/study)^†^	Australia (AusDiab, NHF‐RFPS, WPHC), Austria, Germany (MONICA Augsburg, PROCAM), NZ (PREDICT), Scotland (Renfrew/Paisley, SHHEC), UK (CPRD, QResearch, SABRE, THIN), US (NHANES)	Australia (AusDiab, WPHC), Austria, China, Germany (DETECT), Ireland/France (PRIME), Italy, Japan, Malaysia (NHMS), Netherlands, UK (EPIC‐Norfolk, THIN), US (MCB, MESA, NHANES)	None	QRISK2: Estonia (Biobank), Italy, Netherlands, UK (CPRD, SABRE, THIN), US (MCB) QRISK3: Japan	UK (CPRD, QResearch, THIN)	Australia (NHF‐RFPS), Austria (VHM&PP), China, Estonia (Biobank), Italy, Japan, Malaysia (NHMS), Netherlands (Hoorn), Norway, Spain, US (ARIC, NHANES)	Norway (CONOR)	Australia (AusDiab), Austria, China (PAR), Estonia (Biobank), Germany (DETECT), Japan, Korea (KHS), Malaysia, NZ (PREDICT), US (BioImage, MCB, MESA, REGARDS)	Japan (SHCG)	Validated in 19 external cohorts (14 APCSC cohorts, CMCS, HCUR, PREDICT, TLGS, UK Biobank); nations include Australia, China, Iran, Japan, NZ, Singapore, Thailand, UK
Model performance in Australian cohort	AusDiab (10‐year risk): women: Brier score, 0.028; C‐statistic, 0.80 (0.76–0.84); men: Brier score, 0.078; C‐statistic, 0.74 (0.71–0.78) NHF‐RFPS: AUROC, 0.86 (0.79–0.93); HL χ^2^, 4.74 (*P* = 0.192) WPHC (5‐year risk): C‐statistic, 0.67 (0.64–0.70); Cook χ^2^, 43.84 (*P* < 0.001) WPHC (10‐year risk): C‐statistic, 0.671 (0.643–0.699); Cook χ^2^, 65.91 (*P* < 0.001)	AusDiab (10‐year risk: traditional model): women: Brier score, 0.029; C‐statistic, 0.82 (0.79–0.86); men: Brier score, 0.084; C‐statistic, 0.76 (0.73–0.79) WPHC (10‐year risk): C‐statistic, 0.67 (0.64–0.70) Cook χ^2^, 116.13 (*P* < 0.001)				NHF‐RFPS High risk score: AUROC, 0.88 (0.83‐0.93); HL χ^2^, 12.06 (*P* = 0.007) Low risk score: AUROC, 0.88 (0.83–0.93); HL χ^2^, 6.09 (*P* = 0.107)		AusDiab (10‐year risk) Women: Brier score, 0.027; C‐statistic, 0.84 (0.80–0.87); men: Brier score, 0.076; C‐statistic, 0.77 (0.74–0.80)		APCSC Laboratory model: C‐index, 0.755 (0.726–0.784) Non‐laboratory model: C‐index, 0.753 (0.724–0.782)
Population representativeness (based on derivation dataset)	Data from one region in US, not representative	Data from one region in US, not representative	Nationally representative cohort — electronic primary care records	Nationally representative cohort — electronic primary care records	Nationally representative survey	Predominantly large representative population‐based cohorts (3/12 cohorts were men‐only); overall large sample likely to be broadly representative	Development dataset based on single survey (58% participation rate); unlikely to be representative of broader Norwegian population (risk scores likely underestimate risk in general population)	Several community‐based, racially diverse, cohort studies; low numbers overall (24 000 participants) and small number of African American participants (unlikely to represent this group)	Large population‐based cohort study in urban Japanese population; unlikely to be representative of whole Japanese population	Largely derived from high income countries in Emerging Risk Factors Collaboration; does not capture variation in CVD risk within subregion or countries
Comments/developments				Updated/calibrated annually; equations include interaction terms between several variables		Original publication produced separate charts for each cholesterol measure (either TC or TC:HDL‐C); separate charts used for high risk and low risk European countries; classification based on age‐adjusted 2012 CVD mortality rate cut‐off points	Different surveys from those used in model development	2019 guidelines recommend CAC measurement (and consideration of other risk‐enhancing factors) to reclassify intermediate/borderline risk adults	Separate equations for each cholesterol measure (either LDL‐C or TC)	Data included two small scale Australian studies (Abdominal Aortic Aneurysm Screening Program, and Busselton)

References: Framingham–Anderson (1991);^4^, ^6^, ^34^, ^37^, ^43^, ^44^, ^45^, ^46^, ^47^, ^48^, ^49^, ^50^, ^51^, ^52^, ^53^, ^54^, ^55^, ^56^ Framingham–D'Agostino (2008);^6^, ^32^, ^51^, ^53^, ^54^, ^55^, ^56^, ^57^, ^58^, ^59^, ^60^, ^61^, ^62^, ^63^, ^64^, ^65^, ^66^, ^67^ PREDICT;^33^ QRISK2/QRISK3;^34^, ^35^, ^48^, ^49^, ^52^, ^57^, ^58^, ^59^, ^60^, ^68^ ASSIGN;^21^, ^37^, ^47^, ^52^, ^53^ SCORE;^23^, ^38^, ^50^, ^54^, ^58^, ^60^, ^61^, ^62^, ^68^, ^69^, ^70^, ^71^, ^72^, ^73^ NORRISK 2;^39^ Pooled Cohort Equations;^26^, ^27^, ^33^, ^40^, ^55^, ^56^, ^59^, ^60^, ^61^, ^63^, ^64^, ^65^, ^68^, ^74^, ^75^, ^76^, ^77^, ^78^ Suita score;^28^, ^41^, ^79^ WHO risk charts (2019).^29^, ^42^

AF = atrial fibrillation; APCSC = Asia Pacific Cohort Studies Collaboration; ARIC = Atherosclerosis Risk in Communities study; AUROC = area under receiver operating characteristic; AusDiab = Australian Diabetes, Obesity and Lifestyle Study; BMI = body mass index; BP = blood pressure; CABG = coronary artery bypass graft; CAC = coronary artery calcium; CHD = coronary heart disease; CHF = congestive heart failure; CKD = chronic kidney disease; CMCS = Chinese Multi‐Provincial Cohort Study; Cook χ^2^ = Cook chi‐squared goodness‐of‐fit test; CPRD = Clinical Practice Research Datalink; DBP = diastolic blood pressure; DETECT = Diabetes and Cardiovascular Risk Evaluation: Targets and Essential Data for Commitment of Treatment (study); DM = diabetes mellitus; EPIC‐Norfolk = European Prospective Investigation of Cancer‐Norfolk (cohort); HCUR = Health Checks Ubon Ratchathani (study); HDL‐C = high‐density lipoprotein cholesterol; HL χ^2^ = Hosmer‐Lemeshow chi‐squared goodness‐of‐fit test; KHS = Korean Heart Study; LDL‐C = low‐density lipoprotein cholesterol; LVH = left ventricular hypertrophy; MCB = Mayo Clinic Biobank; MESA = Multi‐Ethnic Study of Atherosclerosis; MI = myocardial infarction; MONICA = Monitoring trends and determinants in cardiovascular disease (project); NHANES = National Health and Nutrition Examination Survey; NHF‐RFPS = National Heart Foundation – Risk Factor Prevalence Study; NHMS = National Health and Morbidity Survey; PAD = peripheral artery disease; PAR = Prediction for ASCVD Risk in China (project); PRIME = Prospective Epidemiological Study of Myocardial Infarction; PROCAM = Prospective Cardiovascular Münster (study); PTCA = percutaneous transluminal coronary angioplasty; PVD = peripheral vascular disease; RA = rheumatoid arthritis; REGARDS = REasons for Geographic and Racial Differences in Stroke (study); SABRE = Southall And Brent REvisited (cohort); SBP = systolic blood pressure; SHCG = Specific Health Check and Guidance in Japan (project); SLE = systemic lupus erythematosus; T1DM = type 1 diabetes mellitus; T2DM = type 2 diabetes mellitus; TC = total cholesterol; THIN = The Health Improvement Network (database); TIA = transient ischaemic attack; TLGS = Tehran Lipids and Glucose Study; VHM&PP = Vorarlberg Health Monitoring and Promotion Programme; WHO = World Health Organization; WPHC = Well Person's Health Check (cohort).

* Table adapted from 2016 European guidelines on cardiovascular disease prevention in clinical practice.^23^

† Only includes external validation of original risk equations (recalibrated and country‐adapted equations not included).

## Evaluation of CVD risk equations against the selection criteria

Overall, the identified equations varied in the extent to which they met the selection criteria (Box 4). The NZ PREDICT equation and UK QRISK3 equation most closely met the selection criteria, with PREDICT meeting seven and QRISK3 meeting six of the eight criteria.

Box 4 **Assessment of cardiovascular disease (CVD) risk equations against selection criteria**

**Table:** 

Selection criteria	Framingham–Anderson (1991)	Framingham–D'Agostino (2008)	PREDICT	QRISK2/QRISK3	ASSIGN	SCORE	NORRISK 2	Pooled Cohort Equations	Suita score	WHO risk charts (2019)
Contemporary data sources (includes data from past 10–20 years)	✘	✘	✓	✓	✘	✘	* (data from 1994–2009)	✘	✘	* (data from 1960–2013)
Established CVD risk factors	✓	✓	✓	✓	✓	✘ (diabetes not included in equation)	✘ (diabetes not included in equation)	✓	✓	✓
Measures of ethnicity and social deprivation	✘	✘	✓	✓	* (includes deprivation but not ethnicity)	✘	✘	* (includes ethnicity but not deprivation)	✘	✘
Broad CVD (events and deaths) outcomes	✓	✓	✓	✓	✓	✘	✘ (predicts acute myocardial infarction and cerebral stroke)	✓ (focus on hard end points)	✘	✓
Population representativeness	✘	✘	✓	✓	✓	✓	✘	*	✘	✘
External validation in populations similar to that of Australia	✓	✓	✘	✘	✘	✘	✘	✓	✘	✓ (validated in dataset which included some Australian data)
Ability to be recalibrated and modified	✘ (can possibly be recalibrated but no representative data on left ventricular hypertrophy)	* (can be recalibrated but not modified without access to data sources)	✓ (can be modified and recalibrated for CVD incidence and some risk factor data by direct comparison)	✘ (no appropriate data to predict risks)	✘ (no data on family history to predict risks)	* (can be recalibrated but not modified without access to data sources)	✘ (no appropriate data to predict risks)	✘ (no appropriate data to predict risks)	* (can be recalibrated but not modified without access to data sources)	* (can be recalibrated but not modified without access to data sources)

✓ = meets selection criterion in full;

* = partially meets selection criterion; ✘ = does not meet selection criterion; WHO = World Health Organization.

**Contemporary data sources.** Only the PREDICT and QRISK2/QRISK3 equations include data primarily published in the past 20 years, with PREDICT including data from 2002–2015 and QRISK3 using data from 1998–2015 (Box 3). The WHO risk charts also use some more recent data, with baseline data from 85 prospective cohorts covering the period 1960–2013 (Box 3). All other risk equations used data from the 1960s through to the 1990s.

**Inclusion of established CVD risk factors.** All the risk equations captured the established CVD risk factors of age, sex, smoking, diabetes, blood pressure and cholesterol, except SCORE and NORRISK 2, which considered diabetes a clinically determined high risk criterion (Box 3). Although not specifically assessed in this review, there is also a diabetes‐specific version of the PREDICT equation.^80^

**Ethnicity and social deprivation.** Both the PREDICT and QRISK2/QRISK3 equations include ethnicity and social deprivation as risk predictors (Box 3 and Box 4). The Scottish ASSIGN equation includes deprivation but not ethnicity, while the US Pooled Cohort Equations include race but not social deprivation (Box 3 and Box 4).

**Global CVD events and deaths outcomes.** All the CVD risk equations predict, at a minimum, coronary heart disease (CHD) and stroke events and deaths, except for the Suita score, SCORE and NORRISK 2 equations (Box 3 and Box 4). The Suita score predicts CHD events and deaths but not stroke; SCORE predicts fatal CVD outcomes only; and NORRISK 2 only predicts acute myocardial infarction (MI) and stroke (Box 3). The Framingham–Anderson (1991), Framingham–D'Agostino (2008) and PREDICT equations had the most global CVD outcomes, including unstable angina, peripheral vascular disease, transient ischaemic attack (TIA) and heart failure, in addition to other CHD and stroke (Box 3). QRISK3 predicts 10‐year risk of CHD (angina and MI), ischaemic stroke and TIA (Box 3).

**Population representativeness.** PREDICT, QRISK2/QRISK3, ASSIGN and SCORE were developed using large scale development datasets that are likely to be broadly representative of their target populations (Box 3). While these four risk equations are considered to be broadly representative of their target populations, only PREDICT and QRISK2/QRISK3 were developed using electronic health records and are largely representative of the primary care populations in these countries. ASSIGN and SCORE were based primarily on population‐based cohort and survey datasets covering large proportions of their respective populations.

**Excellent model performance.** Measures of discrimination for the risk equations in their development datasets are provided in Box 3 but cannot be directly compared between equations as they are applied in different populations. As there are no readily translatable statistics that can be used to compare model performance between risk equations, we did not include this selection criterion within the summary in Box 4. However, many of the risk equations, including PREDICT, QRISK2, ASSIGN, Suita score and Framingham–D'Agostino (2008) have been directly compared with previous risk equations (usually versions of the Framingham equations) within the same population and found to have better model performance for their target populations.

**External validation in populations similar to that of Australia.** Five risk equations have had their performance examined in Australian populations: Framingham–Anderson (1991), Framingham–D'Agostino (2008), SCORE, Pooled Cohort Equations and the WHO risk charts (Box 3 and Box 4). The performance of the Framingham–Anderson (1991) equation has been examined in three Australian cohorts, with small sample sizes in each: the Australian Diabetes, Obesity and Lifestyle Study;^6^, ^56^ the Well Person's Health Check;^51^ and the National Heart Foundation Risk Factor Prevalence Study^50^ (Box 3). Within these limitations, the equations performed reasonably well in terms of discrimination and recalibration for the general Australian population, but underestimated risk in Aboriginal and Torres Strait Islander populations (Well Person's Health Check) (Box 3). It should be noted that these studies examined the performance of the equation alone, without the application of clinical criteria placing people automatically at high risk. Neither the PREDICT nor QRISK2/QRISK3 equations have been examined in an Australian population.

**Recalibration potential.** Of the 10 CVD risk equations evaluated, five could potentially be recalibrated: Framingham–D'Agostino (2008), PREDICT, SCORE, Suita score, and the WHO risk charts (Box 4). All these equations, except PREDICT, could be recalibrated using CVD event rate and risk factor data from national health surveys.

## Usability and practical implications

Due to the number of risk factors included in the PREDICT and QRISK3 risk equations, neither can practically be applied through colour risk charts, which are useful in situations where appropriate electronic resources are not available. Both PREDICT and QRISK3 were generated using data from primary care encounters and can only be implemented in practice using an online risk calculator, or risk calculators embedded within general practice software. All the risk factors in PREDICT could feasibly be measured and recorded during normal primary care consultations in Australia. However, the number of risk factors and the detailed information needed on other medical conditions and medications in QRISK3 means that it would be difficult to apply in practice in Australia. QRISK3 was designed specifically for use in the UK and is integrated within electronic health records such that risk factor data can be prefilled automatically using information recorded in a patient's electronic record. The UK also uses a standard classification system for disease diagnoses, which means that these are captured in the same way across general practice systems. This is often not the case in Australia. These differences mean that it is currently not possible to practically implement QRISK3 in the Australian primary care system.

## Discussion

The NZ PREDICT and UK QRISK3 equations most closely matched the agreed criteria for selecting a new CVD risk prediction equation for Australia. Both were developed using contemporary representative datasets, were derived for use in primary care settings, and incorporate data from established CVD risk factors and additional clinical risk predictors. PREDICT met the considered usability requirements and was found to be the international equation that combined meeting the most selection criteria with the ability to be directly modified, due to open availability.

While measures of ethnicity and social deprivation can help account for differences in observed CVD incidence (often acting as proxies for other causal factors), in practical terms, existing categories in international risk equations can be difficult to translate to the Australian context, unless there is the ability to modify the risk equation to change underlying measures or categories. PREDICT was the only CVD risk equation that could be directly modified to adjust ethnicity and deprivation measures to be more suitable to Australia.

A key limitation of adopting the PREDICT equation in Australia is that its performance has not been, and currently cannot be, directly examined in an Australian dataset owing to a lack of datasets containing all the necessary risk factors and outcomes data. However, PREDICT has been directly compared with previous Framingham equations in NZ and found to perform better. As Australia's population is more like the NZ population than a selected 1970s predominantly white US population (used to develop the Framingham equations), it is very likely that CVD risk prediction would be improved by using PREDICT rather than the Framingham equation, and recalibrating it for use in the Australian population. Priority should be given to validating the PREDICT equation within Australia when appropriate data become available.

CVD risk equations integrated into general practice software are more likely to be taken up in practice. In Australia, there are several software vendors supplying decision support tools to primary care. Ideally the CVD risk equation used in Australia will be integrated consistently into existing general practice software, with fields being pre‐populated from clinical records.

The CVD risk equations assessed in our review were all from high income nations, and other than Australia, Japan, NZ and the global WHO risk charts, were solely from North America and Europe. There is an under‐representation of equations from other global regions, such as Asia, reflecting in part a lack of major guidelines or, in the case of Chinese guidelines for CVD prevention,^81^ a lack of accessible information on the risk equation used.

Since undertaking this review, updated versions of both the Canadian^11^ and European^22^ guidelines have been published (both in 2021). We did not assess these latest guidelines as both were published after completion of the current review and were not available in time to inform the updating of the Australian CVD risk assessment and management guidelines. There were no changes to the risk prediction equation recommended in the Canadian guidelines; however, the European guidelines now recommend the use of SCORE2,^82^ an updated version of SCORE. SCORE2 predicts both fatal CVD and non‐fatal MI and stroke, and now includes diabetes in the equation. SCORE2 would have met three of the selection criteria, thereby not changing the overall conclusions of this review.

In Australia, efforts should be made to develop the infrastructure needed to assimilate data from different primary care software platforms and link with hospital and deaths data, such that an Australian‐specific CVD risk equation can be developed.

## Conclusion

We used a systematic approach to review evidence on existing international CVD risk equations and compare them to a set of a priori defined selection criteria developed for Australia. The NZ PREDICT equation met the greatest number of selection criteria and was considered most feasible to implement in Australian primary care. The PREDICT equation, which was developed in a contemporary, diverse primary care population and includes measures of social deprivation, is likely to offer better detection of CVD risk in Australia compared with the currently used Framingham risk equation. There needs to be careful consideration of how to implement the PREDICT equation in Australian primary care to ensure use is in accordance with guideline recommendations across different general practice software platforms. Ultimately, Australia should be working towards capturing and linking the data necessary to develop Australian‐specific CVD risk equations that can be refined and updated over time.

## Open access

Open access publishing facilitated by Australian National University, as part of the Wiley ‐ Australian National University agreement via the Council of Australian University Librarians.

## Competing interests

No relevant disclosures.

## Provenance

Not commissioned; externally peer reviewed.
